# Statistical Pass-By for Unattended Road Traffic Noise Measurement in an Urban Environment

**DOI:** 10.3390/s22228767

**Published:** 2022-11-13

**Authors:** Elena Ascari, Mauro Cerchiai, Luca Fredianelli, Gaetano Licitra

**Affiliations:** 1 Institute for Chemical-Physical Processes of the Italian Research Council (CNR-IPCF), Via Giuseppe Moruzzi 1, 56124 Pisa, Italy; 2 Environmental Protection Agency of Tuscany Region, Pisa Department, Via Vittorio Veneto 27, 56127 Pisa, Italy

**Keywords:** SPB method, sound pass-by, low-noise surfaces, noise modeling, road traffic noise, unattended noise measurement procedure, traffic measurements, noise emission, environmental noise, sound

## Abstract

Low-noise surfaces have become a common mitigation action in the last decade, so much so that different methods for feature extraction have been established to evaluate their efficacy. Among these, the Close Proximity Index (CPX) evaluates the noise emissions by means of multiple runs at different speeds performed with a vehicle equipped with a reference tire and with acoustic sensors close to the wheel. However, signals acquired with CPX make it source oriented, and the analysis does not consider the real traffic flow of the studied site for a receiver-oriented approach. These aspects are remedied by Statistical Pass-By (SPB), a method based on sensor feature extraction with live detection of events; noise and speed acquisitions are performed at the roadside in real case scenarios. Unfortunately, the specific SPB requirements for its measurement setup do not allow an evaluation in urban context unless a special setup is used, but this may alter the acoustical context in which the measurement was performed. The present paper illustrates the testing and validation of a method named Urban Pass-By (U-SPB), developed during the LIFE NEREiDE project. U-SPB originates from standard SPB, exploits unattended measurements and develops an in-lab feature detection and extraction procedure. The U-SPB extends the evaluation in terms of before/after data comparison of the efficiency of low-noise laying in an urban context while combining the estimation of long-term noise levels and traffic parameters for other environmental noise purposes, such as noise mapping and action planning.

## 1. Introduction

The “Noise in Europe 2020” report [1] by the European Environment Agency confirmed that road traffic is the most dominant source of environmental noise, with an estimated 113 million Europeans affected by noise levels greater than 55 dB(A) of L_den_ (day–evening–night level). Moreover, at least 20% of the EU population lives in areas where road traffic noise levels are high, and it is likely that the inhabitants underestimate the well-known long-term effect on health that prolonged exposure can produce.

Among the many different mitigation actions that can be applied, the laying of new low-noise pavement (LNP) [2,3], such as open-graded pavement, rubber asphalts or poroelastic surfaces, has become popular as it significantly affects a wider area and a greater number of citizens compared to other actions focused on a single building. LNP is defined as a road surface which can reduce sound emissions to some extent compared to a reference pavement. The most widely used definition of LNP is “pavement able to provide a reduction of 3 dB(A) with respect to a Dense Asphalt Concrete (DAC)” [4].

Noise barriers have visive impact that reduces their acceptability [5,6], and the installation of soundproof windows is much more expensive in cases where there are multiple receivers to mitigate [6,7]. Moreover, new windows do not completely solve the problem as citizens leave them open in summertime [8].

The laying of LNP is now a common mitigation action in the EU, and a few methods have been established by technical norms that have emerged over the years in order to evaluate the performances of road surfaces. Among these, the Close Proximity Index (CPX), according to ISO 11819-2:2017 [9], the Statistical Pass-By (SPB-ISO), according to ISO/DIS 11819-1 [10], and the ISO 13472-1:2022 [11], are all non-intrusive methods requiring on-site measurements, unlike ISO 10534-1:1996 [12], which applies in-lab Kundt tube tests to asphalt samples. However, different studies in the past demonstrated that these standard monitoring techniques are not sufficient to reliably evaluate the pavement acoustic efficacy or to directly compare the properties of different ones [13,14].

CPX has been applied and improved over the years, reaching the status of a well-recognized methodology [15,16,17,18], and its indicators are mentioned within the minimum environmental levels given by the EU Green Public Procurement Criteria (GPP), which were finally defined in 2016 for Europe [19]. The quality of new LNP should then be guaranteed by a mandatory evaluation of its noise emission through CPX measurements, as it is the method that is more directly focused on evaluating the source emission by measuring noise with microphones next to the reference tire of a lab-moving vehicle.

On the contrary, SPB-ISO is the right method to identify the sound emitted by real flow or for different vehicle categories, as it is based on noise and traffic data measurements performed at the roadside and at a standard distance. It provides an indicator of noise perceived for a single vehicle and may help to verify the noise efficacy of the pavement or to introduce policies for specific category restrictions or speed limits in contrast to methodologies which acquire the overall noise. Moreover, SPB-ISO is also very helpful for the definition of noise models in the noise mapping and action plan phases. This method is also able to characterize new categories, such as, for example, electric vehicles, which are an emerging issue the definition of which is left open even in the noise mapping official method CNOSSOS-EU [20]. In particular, a variation of the SPB-ISO, the Controlled Pass-By (CPB) [21] is being used in the ongoing LIFE Project E-VIA, aiming to assess the noisiness of electric vehicles on specific-noise pavements [22]. In fact, low-noise pavements can reduce rolling noise; thus, their effect is maximal for electric vehicles [23], whilst it is reduced for heavy vehicles and mopeds. Low-noise surfaces were also recently under study in a combined solution with Intelligent Transportation Systems (ITS) in order to maximize their mitigation effect with more focused traffic [24].

Even though both CPX and SPB-ISO are widely performed around the world [25,26,27], they have been shown to not fit perfectly into the urban environment, especially the standard SPB methodology [13]. In addition, the SILENCE project contributed to the development of the Backing Board variant (ISO 11819-4 [28]), the measurement of which is still influenced by the real world in front of the microphone.

SPB-ISO measurements not only require a free field around the mics, which is rare in an urban context, but also single vehicles passing within a sufficient time spacing. Such a condition almost never happens on urban roads during the daytime, especially on the major and most impactful roads that have high traffic flow. Traffic lights, pedestrian crossing and roundabouts are also urban issues that group vehicles together, especially during daytime, which is the period when attended SPB-ISO should be carried out.

Thus, a method able to determine vehicle noise on road pavements in any urban context was still being looked for when the LIFE NEREiDE [29] project was conceived. The project, among others, also aims to develop innovative measurement protocols and new methods to verify the efficacy of low-impact surfaces to improve soundproofing performances and reduce annoyance in the urban context, such as through the use of P-U sensors [30]. Therefore, the NEREiDE project intends to improve the existing measurement protocol of SPB in order to provide institutions with more reliable data from monitoring campaigns.

The present work presents a methodology to determine average noise levels emitted by vehicles categories in an urban context. The outcome is called Urban Pass-By (U-SPB) and mixes standard long-term noise measurement with the SPB-ISO method. It has the advantages of an easy setup of common instrumentation, and, most importantly, it can be unattended during the data acquisition, thus, sparing man hours. The developed algorithms allow the elaboration of the measurements to obtain a U-SPB index that is comparable with the SPBI set by ISO/DIS 11819-1. Finally, the most important advantage is that this procedure implements statistical pass-by in an urban context, ascribing value to such results for mitigation purposes.

The present paper details the measurement setup and the analysis procedure of U-SPB. The testing and validation phases are based on specific measurement campaigns performed in Tuscany, Italy, and application to monitor the noise performances of new surfaces in an urban context is also shown.

The locally estimated values are not generalizable for all road surfaces, making the U-SPB a method that is not developed enough to label road surfaces over their entire stretch as the CPX does. However, this behavior turned out to be a pro in different contexts, such as the local evaluation of a road noise mitigation action or the evaluation of the effectiveness of traffic reduction measures (times of prohibition of freight transport, bus lines, electric vehicle policies, speed limits, etc.) on the noise impact. This aspect represents an added value for policy makers and allows the correct planning of traffic route policies such as freight-banning times, bus routes, electric vehicle policies, speed limits, etc. The procedure also allows the estimation of long-term L_den_ according to a relation based on measured road traffic flows only. This allows the avoidance of spurious events or unwanted noise sources in the evaluation of people exposed to the noise specifically emitted by the investigated road.

## 2. Background

In 2006, the Tuscany region planned the LEOPOLDO project with the intent to develop innovative noise mitigation techniques for action plans relating to road infrastructure to find the best surface criteria based on the surroundings of the laying and to develop measurement protocols useful for assessing road surface effectiveness and time stability in terms of both acoustical and safety characteristics. SPB-ISO and CPX were applied within the LEOPOLDO project, and improved protocols for measurements and for data postprocessing were developed [31,32]. An improved version of SPB was developed (SPB-L) using a different noise metric than the original version. The SPB-L procedure was based on measuring the acoustical energy of the passing vehicle; it uses the Sound Exposure Level (SEL or L_E_), calculated according to the ISO 1996-1 [33], and the pass-by event is the signal part in which L_Afmax_ exceeds the background noise by more than 10 dB(A), as defined in ISO/DIS 11819-1 [10].

A scientific debate was raised at that time [34,35,36,37], and different metrics were proposed for SPB analysis, which led the LEOPOLDO project to adopt SEL instead of L_Afmax_ as a metric in its SPB-L method. Later, the ROSANNE project [38] found good correlation between L_Afmax_ and SEL. Although SEL is considered more sensitive to ground effects, it is still considered as a relevant option for helping the definition of common noise modeling methods [39].

The SPB-L followed the HARMONOISE [35] and IMAGINE [36] projects, which introduced a second measurement position placed at 3.0 m height and 7.5 m distance from the center of the road lane (SPB-HI). This improved the evaluation of the influence of the local context by avoiding the roadside ground influence that was not negligible with only the 1.2 m height position.

Thus, SPB-L measurements include two microphone heights. Thus, the statistical sample of many single passages constitutes the dataset for the logarithmic regression (Equation (1)) between the measured speed *v* and the *SEL*, which estimates the level at the reference speed *v*_0_, in accordance with SPB-HI.

(1) $$SEL=a+b\cdot log\left(\frac{v}{v_{0}}\right)$$

where *a* is the *SEL* at reference speed (typically 50 km/h), and *b* is a speed-related correction.

A further improvement of SPB-L was the introduction of the use of statistical data binning since the SPB-HI procedure fails when speed data are collected around a single value, such as the speed limit. In fact, the variability due to driver behavior and to vehicle characteristics dominates when the speeds are almost the same. Data then have a cloud-like shape, and those falling outside the cloud influence the fit algorithm. This influence is avoided through statistical data binning applied to the whole dataset and a minimum chi-square fit of central values with their uncertainties.

In more detail, data are grouped in velocity classes, or bins, about 10 km/h in width. The actual width is chosen to minimize the total chi-square of the final fit. The mean and standard deviations of data in each class are computed with the hypothesis of a Poissonian distribution of the vehicles data, with each class represented by the triplet: central velocity of the class and the mean and standard deviation of the SEL data contained in each class. These triplets are used as inputs in the best fit between SEL and speed. In this way, the information of data spread out, and the numerousness in each speed bin is taken into account by means of the uncertainty associated with the central values.

Within the NEREiDE project, a further improvement of SPB was looked for in order to simplify SPB measurement and analysis protocol, especially sparing man time on site. The U-SPB protocol, presented in the present work, intends to derive indexes able to represent pavement efficacy in their implementation context and to derive a method able to identify the contribution that a new pavement might provide in that context, taking into account the fleet composition, average speed and surrounding additional noise sources. 

In Table 1, the characteristics of U-SPB are compared to the different adaptations of the SPB-ISO method in order to clarify the different approaches mentioned in the present work.

## 3. Materials and Methods

An improved SPB methodology that is able to provide similar indicators but avoids the need for the measurements to be attended is defined on the basis of literature review and previous experiences. The methodology described in the next chapter consists of obtaining the pass-by index directly from the noise monitoring station that is usually installed for law requirement purposes. The microphone used for noise level acquisition is placed on the roadside at 4 m height. The single-vehicle passages, required by SPB-ISO, can be easily identified in the noise time histories acquired during the nighttime, when traffic flows and background noise are lower. After its definition, the methodology is finalized according to the following phases:

- Testing, including a feasibility test, performed to confirm the hypothesis, and a controlled comparison with the SPB-L method;
- Validation: comparison of the results obtained by the application of the U-SPB and SPB-L methods;
- Application: within the NEREiDE project, U-SPB is applied to the sites where new low-noise surfaces have been laid in order to evaluate both ante- and post-operam conditions.

Each phase is based on measurement campaigns specifically performed in Tuscany (Italy), which are summarized in Table 2 together with a summary of their most peculiar features.

### 3.1. Testing

The work started with a preliminary analysis of previously gathered data, performed in order to verify the applicability of the main idea. The dataset was retrieved from a noise measurement campaign carried out in SR 439 (Capannori, LU, Italy) for the monitoring of a one-year-old low-noise pavement (ISO 10844 optimized texture, commonly used by the Tuscany region in its road action plan). In this case, the acquisitions were originally performed with a noise monitoring station at the roadside, 10 m from the center lane, in order to verify law requirements in terms of ante- vs. post-operam noise levels. As these data were not acquired ad hoc, a preliminary procedure was applied in order to link night traffic acquired passages with noise levels acquired with a short time interval (50 ms). The pass-by events were identified, and those surpassing the background noise by at least 10 dB(A) in the noise time history were selected. According to vehicle length, categories were established, setting appropriate thresholds for two wheelers and light and heavy vehicles. This preliminary test was intended to verify the feasibility of deriving a model predicting the noise emitted by categories of vehicles by means of fits for speed vs. the different noise metrics (SEL, L_Amax_, L_Aeq_) of a single vehicle passing in front of the measurement station. Distances from the monitoring station to the center lanes were known, and data were corrected for distance using a linear divergence model [37,40] in order to enable comparison with the standard requirements (1.2 m height, 7.5 m distant from center lane). Such a technique is valuable for a comparison at different sites and can provide indexes that are comparable with standard ones. A linear correction is mandatory to compare techniques, i.e., the single moving vehicle passing by has to be represented as a linear source, and divergence rules are applied, as in Equation (2).

(2) $$NM=NM_{meas}+10\log\frac{\sqrt{d^{2}+4^{2}}}{\sqrt{7.5^{2}+1.2^{2}}}$$

where *NM* is the selected noise metrics. 

Once the feasibility was achieved, a procedure was then established for acquiring data. A comparative analysis with the standard method was also performed in order to:

- Define which sound level metric should be fitted with speed (SEL, L_Amax_, L_eq_);
- Verify if categorization with a traffic counter is comparable with a man-made one or if, at least, outliers are not relevant;
- Verify the influence of microphone height and position on the results’ quality and the ability of the instrumentation to detect both lanes.

**Figure 1:**
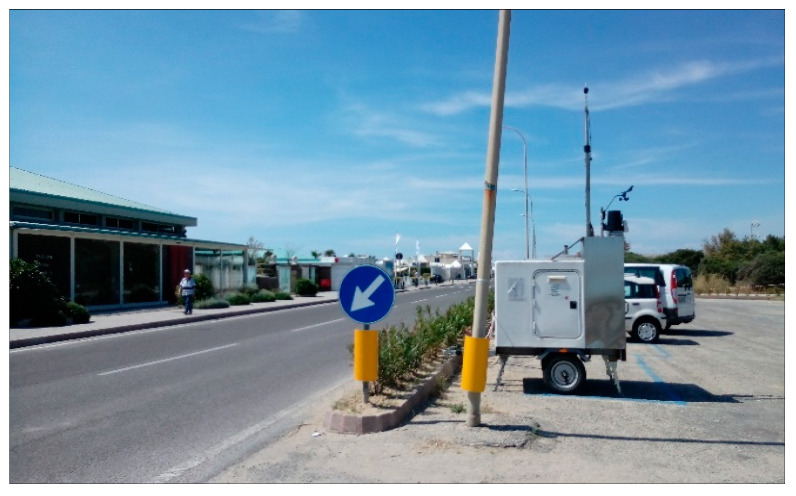
Urban pass-by location in T1 (Marina di Pisa).

**Figure 2:**
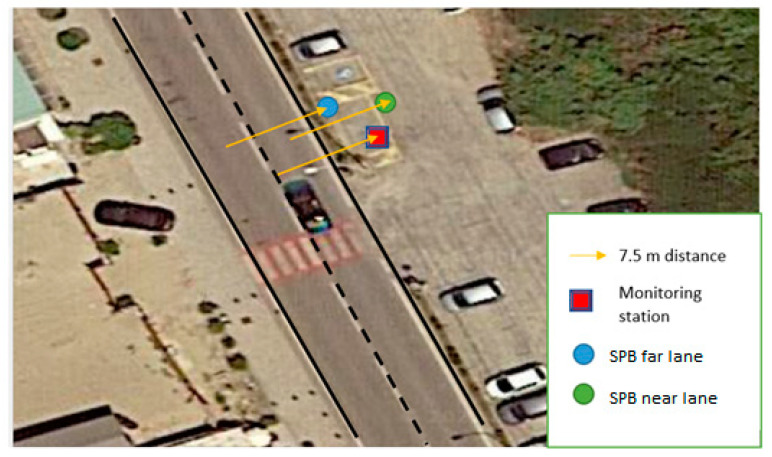
Measurement setup in T1 (Marina di Pisa). Near lane is the one close to the traffic counter and monitoring station; far lane is the one in the opposite direction.

A simple, straight road with free field conditions and a standard pavement (dense asphalt concrete) was selected for this phase. The site was chosen for the performance of both standard SPB and U-SPB in a short time. Figure 1 and Figure 2 report the measurement location and measurement setup as an example.

The test compared the U-SPB with SPB-L on two different sets of data; the first set compared the morning traffic flow sample acquired with SPB-L and at the monitoring station, while the second set compared the SPB morning measured data with nighttime data on the monitoring station, identified by the traffic counter, i.e., applied the U-SPB procedure as detailed in the following chapter.

This phase was also dedicated to demonstrating the feasibility of estimating noise levels at a roadside from an SEL vs. speed model. In fact, a simulated, hourly, A-weighted L_eq_ (L_Aeq,h_) was derived using flows acquired with the traffic counter, and the noise model for SEL was obtained. Namely, the L_Aeq,h_ was estimated according to the following equation:

(3) $$L_{Aeq,h}=\underset{i}{⊕}\left(10\cdot \log\left(Q_{i}\right)+a_{i}+b_{i}\cdot \log\left(\frac{v_{i}}{50}\right)\right)-10\cdot \log\left(3600\right)$$

where the symbol ⊕ means the energetic sum, *Q_i_* is the traffic flow and *v_i_* is the average speed of the *i*^th^ category at the specific hour.

### 3.2. Validation

After the positive outcomes of the testing phase, a validation on low-noise pavements was carried out to increase the statistics of the U-SPB application. The objective of the validation was to confirm the validity of the methodology results through a comparison with other standard on-site methods and to provide a definitive protocol for the urban pass-by methodology, including measurements, a to-do list and analysis guidelines. This phase was carried out by applying the complete protocol to a new area where a mitigation was in force and where the local urban configuration also allowed a full standard application of SPB-L. On the chosen sites, SPB-L, U-SPB and noise equivalent level measurement methods were tested. Noise level estimation through the SEL models was performed and compared with a standard measurement technique in the validation phase.

### 3.3. Application

Finally, the procedure was applied to 4 other pavements in SR 439, corresponding to the NEREiDE project ones. This meant applying the procedure to the data acquired during the ante- and post-operam campaigns and deriving U-SPB values and verifying noise level estimations over a long-term campaign. While the NEREiDE project studied a total of 12 stretches, the present paper only considered the stretches referenced as nos. 1, 3, 5 and 6 in the project [41].

## 4. U-SPB Procedure

The applied U-SPB measurement procedure is derived from the SPB-L experimental protocol.

The procedure intends to combine data from a traffic counter and a standard monitoring station to catch single passages, as in the SPB-L. Then, the statistical data binning is applied and data fitted according to the procedure followed in the LEOPOLDO project, and the SPB index (SPBI) is derived accordingly.

### 4.1. Measurement Setup

The monitoring station includes a microwave traffic counter system, a sound level meter, a power system and a weather station to exclude periods with a wind speed higher than 5 m/s or rainy periods. The traffic counter acquires single-vehicle passage data, such as vehicle length, velocity, transit time and time distance from the previous vehicle passage. The devices should be carefully synchronized in time in order to simplify analysis. The sound level meter should fulfill the ISO 1996-2 requirements and should be positioned preferably 7.5 m away from the centerline. If impossible, the distance from the roadside should not be less than 3 m in order to avoid source directivity problems and be no more than 15 m away in order to avoid ground and spurious reflection issues. In this way, the sight angle of the road is maximized without going too far from centerline and avoiding measuring a lower sound signal. 

The sound meter level should be placed at 4 m height and should be set to acquire:

- L_Aeq_, in fast 50 ms;
- Third-octave band spectrum.

The microwave traffic counter should be installed at the roadside, positioned in order to acquire traffic data of all lanes, and should be set to acquire vehicle length accurately in order to identify the different vehicle categories.

The sound level meter and the traffic counter should be positioned as close to each other as possible in order to avoid speed differences between the acquisitions.

A brief on-site observation is suggested at the beginning of the setup to fix category length limits for both lanes, thus, minimizing category mismatching.

A fixed length for the measurement is not fixed and depends on the flow, but a two-night duration is generally enough to obtain a sufficient number of transits.

### 4.2. Data Analysis

The analysis procedure starts from the raw data obtained in the measurement phase by matching traffic counter flows with noise data (sensor acquisition phase). Measurement periods are filtered according to the national law requirements for weather conditions.

The feature detection phase starts with downloading traffic counter data into a spreadsheet. Then, a time interval between consecutive passages has to be fixed to identify valid transits. The choice of the time interval depends on the source distance and source average speed. For the purpose of this study, a time interval between 3.5 and 4 s is applied since all the roads have 50 km/h as a speed limit. The candidate transits are highlighted by conditional formatting on the spreadsheet. Analogous conditional formatting is performed over vehicle lengths returned by the traffic counter to discriminate between vehicle categories. Then, single candidates are identified in the time history of sound pressure level, and events are selected if L_Afmax_ exceeds the background noise by at least 10 dB(A); otherwise, the event is discarded.

**Figure 3:**
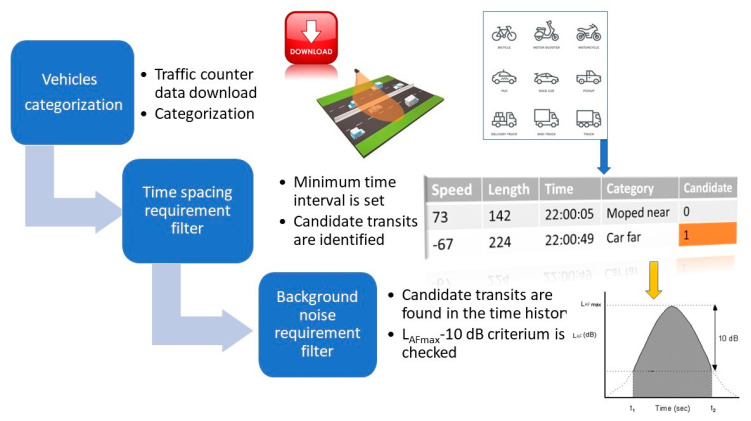
Features extraction phase: event selection process.

For valid events, the noise average spectrum is acquired in the L_Afmax_–10 dB(A) time interval. The analysis spreadsheet is designed to be able to count valid events per direction and categories so that it is possible to stop analysis as soon as the minimum requirements are fulfilled. A diagram of the event selection procedure is reported in Figure 3.

The event extraction phase starts by collecting the average spectrum together with speed and direction and storing them in order to collect all the events. The average spectrum is then fitted according to Equation (1).

**Figure 4:**
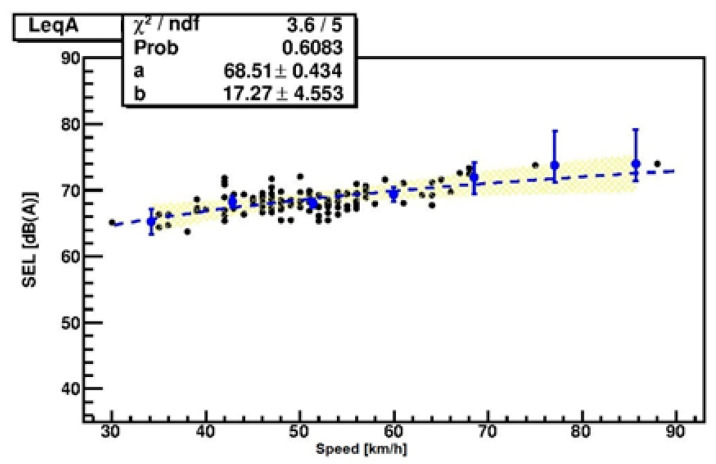
Example of data collected in binning chunks (blue points with error bars) for the best fit between SEL and velocity. The 95% confidence interval from the fit is reported in yellow.

Thus, the statistical sample of many single passages constitutes the dataset for the statistical data binning developed in SPB-L. In Figure 4, an example of statistical data binning is provided according to Zei et al. [42] and Lafferty et al. [43].

The analysis with data binning provides regression of SEL values and each frequency band as a function of speed. An example is reported in Figure 4. In case of fits with fewer than 10 events, fits are carried out without statistical data binning.

If enough events are acquired, at least 100 light and 30 heavy vehicles, the SPBI can be estimated according to Equation (3), taken from the ISO/DIS 11819-1 for low-speed roads:

(4) $$SPBI=\;10\;\log\left[W_{1}10^{\left(\frac{L_{1}}{10}\right)}+W_{2}\left(\frac{v_{1}}{v_{2}}\right)10^{\left(\frac{L_{2}}{10}\right)}+W_{3}\left(\frac{v_{1}}{v_{3}}\right)10^{\left(\frac{L_{3}}{10}\right)}\right]\;$$

where *L*_i_ is the vehicle sound levels for the *i*^th^ vehicle categories; *W*_i_ is the weighting factors determined by compositions of traffic flow and are established by the ISO.

## 5. Results

The present chapter describes the obtained results in the different phases of testing, validation and application of the U-SPB method.

### 5.1. Testing

The feasibility test provided results for SEL, L_Aeq_ and L_Amax_. The pros and cons of each of the metrics are briefly described:

- SEL was chosen by the NORDTEST method, HARMONOISE, IMAGINE and the Italian LEOPOLDO projects for its representativeness in noise models and its ability to estimate the overall noise levels. A potential weakness is represented by its partial dependence on the 10 dB(A) cut made by the operator [41];
- On the contrary, L_Aeq_ is weakly dependent on the 10 dB(A) cut, but it is representative of average vehicles only when their speed variation is small and when the time of passage is almost the same for all vehicles. These conditions are especially difficult to achieve at nighttime, which is when data are intended to be collected, because single-passage events are more frequent;
- L_Amax_ is the ISO/DIS 11819-1 parameter and does not depend on cut, but it is strongly dependent on the local peculiarities of the road (such as potholes, bumps, etc.), which lack homogeneity, and vehicle discrepancies. Thus, its results tend not to be stable even for the same vehicle and pavement.

The data analysis revealed a relevant difference between fits performed by plotting speed against SEL, L_Aeq_ (recomputed by the average spectrum) and L_Amax_ (from spectrum on maximum) for the light vehicle category. 

Then, the model in Equation (4), which is a generalization of Equation (2), with f being one of the three tested parameters, was applied to the measured dataset:

(5) $$f=a+b\cdot log\left(\frac{v}{50}\right)$$

Table 3 shows the results obtained in T0 (Capannori) for each lane and fit values for the light vehicle category. As mentioned in the previous chapter, the near lane direction was the lane closest to the noise monitoring station, and the far lane was the opposite one. Distances from microphones to center lanes were known, and data were corrected for distance using a linear divergence model in order to allow comparison with the standard requirements (1.2 m height, 7.5 m distant from center lane) and between lanes.

The SEL slope results were lower than the other metrics, probably because they already considered time/speed effect. The near lane was noisier according to L_Amax_ and L_Aeq_, while it was not for SEL.

The preliminary test also showed that pass-by parameters can be derived with good fit agreement in terms of $\chi^{2}\;$by starting from data acquired with the monitoring station, and the noisiness of a single vehicle category can be determined as a function of speed.

Then, the testing measurement in T1 (Marina di Pisa) allowed a comparison of the SPB-L and the U-SPB applied over the same sample flows. The test intended to address whether there were differences due to the microphones’ positions, regardless of the speed and sample variability. The three parameters were once again tested. U-SPB data were grouped into the morning common sample, which was intended to be the passages collected on the monitoring station at the same time as the attended SPB-L, and the night independent sample.

Table 4 reports the results for the light vehicle category for each indicator in the first U-SPB dataset. Linear correction for distance was applied to the monitoring station and to the 3 m microphone to allow comparisons.

**Figure 5:**
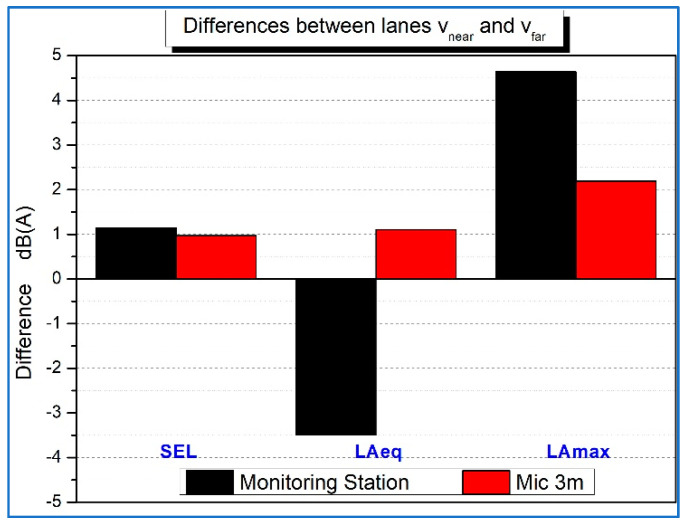
Difference between near and far lanes results with different parameters in T1.

Some relevant differences emerged for each microphone, with lower values for the near lane and higher values for the far lane from the 1.2 m SPB-L microphone. This might be due to the specific location, which presented a small, green hedge at the roadside that might have partially screened noise from the near lane at low heights. The far lane might have been noisier because of some bumps and because of a more reflective pavement than the one in the parking area where the microphones for the near lane were located. Moreover, the levels for all metrics at the 3 m microphone and at the monitoring station were more similar between the lanes, as they were not as affected by ground reflections as the standard microphone at 1.2 m height. As reported in Figure 5, the acquisitions with the microphone at 3 m and with the station were compared in terms of the difference between the two lanes. L_Amax_ was greatly different between lanes for both microphones, confirming that L_Amax_ is more sensitive to local bumps than other indicators and is highly dependent on microphone position. Thus, the L_Amax_ was discarded for its sensitivity to local issues. L_Aeq_ difference highly depends on microphone position due to the duration of the event, which can vary significantly with the sight angle. As expected, the SEL difference was similar at both microphones.

SEL, as from Equation (1), can also be used to estimate the annual averaged A-weighted equivalent sound levels L_Aeq_ from the annual averaged traffic flow, as explained in Equation (2). This represents an additional advantage that should drive the sound pass-by methodology to be optimized for this indicator. On the other hand, SEL depends on time interval above 10 dB(A), while L_Aeq_ is not corrected by the time length of the passage. Thus, as well as being corrected by the distance, the two lanes were differently influenced by the time length for the L_Aeq_ indicator. The greater difference with this indicator resides in the directivity of the source and the geometry of the measurement setup; as the acquisition was next to the source, a shorter time interval was needed to identify a 10 dB(A) cut compared to an acquisition at a greater height from the ground. Thus, the SEL is the one with less influence on position in general, and it can be considered as more reliable. As reported in Table 5, the fits’ parameters of the night dataset with SEL were compared with SPB-L for the three vehicle categories. Table 6 reports the number of events considered for the fits during one-night acquisition for light vehicles and two-night acquisition for the other categories.

The differences between SPB-L and U-SPB appear to be mainly due to the sample when statistics were low, but a good agreement was achieved for light vehicles. Some differences could be due to category mismatching, especially mismatching in identification of small trucks (potentially long light vehicles) and short light vehicles (potentially two wheelers). 

**Figure 6:**
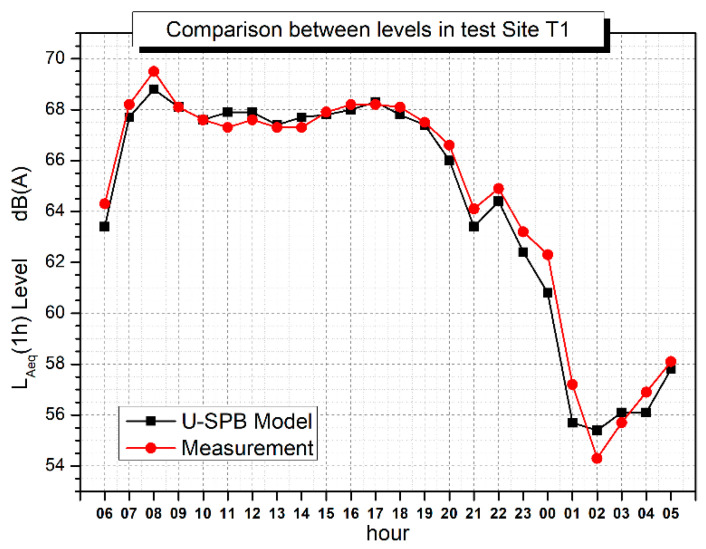
U-SPB L_Aeq,h_ model and measured L_Aeq,h_ comparison in T1.

Even though the measurement period was very short, a model was built to compare measured hourly L_Aeq_ with estimated ones according to Equation (1) (calculated with the night dataset). Figure 6 reports the estimate obtained with U-SPB and measurement over a 24 h monitoring period. The results show that the model is in very good agreement with the measurement, confirming the possibility of estimating noise levels with U-SPB. In particular, the calculated equivalent noise level for reference period day–evening–night L_den_ and for night period L_night_ resulted in values only 0.4 dB(A) and 0.7 dB(A) lower than the measured data, respectively.

### 5.2. Validation

The validation phase was performed in V (Sesto Fiorentino), where a long, straight road section with a free field condition was available to correctly perform SPB measurement with a suitable sample for a daytime, attended section. The road was paved with a low-noise surface. The number of acquired events is reported in Table 7. A single night acquisition was needed for U-SPB analysis of light vehicles and two nights for the heavy and two wheeler categories. A further differentiation of heavy vehicles into two-axis and multi-axis vehicles is reported since it came out that such a distinction improved the result on this road.

The identification of truck classes at the monitoring station was performed based on vehicle length, a procedure that can give some unfortunate mismatching. This further differentiation led us to consider a further category and to compare SPB-L with only two-axis trucks of U-SPB since the number of multi-axis vehicles in the SPB-L sample was lower. Table 8 shows the fit results according to Equation (2).

The two methods’ results were equivalent in terms of the A parameter, apart from the heavy vehicle category in the far lane. Some buses were included in the SPB sample that were not clearly identified in the U-SPB one. 

With all the information, it was possible to estimate the SPBI with Equation (3), calculating for a low-speed road, i.e., with a reference speed equal to 50 km/h, as reported in Table 9.

SPBI values results were equivalent, with a less than 1 dB(A) difference, even if associated uncertainties were high due to the low number of heavy vehicle events. Thus, the procedure can also be considered as validated on a low-noise pavement.

**Figure 7:**
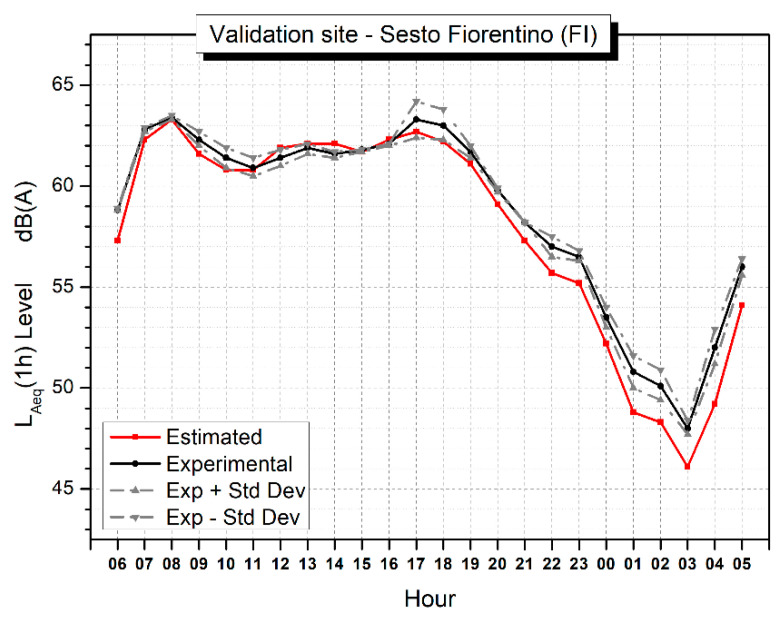
U-SPB L_Aeq,h_ model and measured L_Aeq,h_ comparison in V.

Finally, the authors decided to also verify the coherence between pass-by and noise level measurements, including for the heavy vehicle category. An estimation based on flows was performed using the estimated SEL of each category; heavy vehicle categories were considered using the average SEL of two-axis and multi-axis trucks and the summed flow. The estimated level was compared with the measurement, as shown in Figure 7; while the U-SPB model underestimated the night levels due the influence of other sources (such as birds, roadside fluorescent lamps, etc.), on the measured ones, a substantial equivalence was achieved during the day between the estimated and measured experimental levels, as for the test in T1 (SP 224).

The comparison of noise equivalent level indicators estimated with measurement (hereafter L_meas_) and the pass-by model according to Equation (1) (L_model_) is reported in Table 10. Apart from night levels, when other sources might be relevant, the model was an efficient way to estimate noise levels, with prediction always being slightly lower than measured, as expected.

### 5.3. Application

The measurement procedure was then applied on LIFE NEREiDE sites, where six different pavements (named stretches 1 to 6) were implemented, and their effects in terms of local fleet (ante- and post-operam values) were evaluated in the project. U-SPB was used to solve the problem while it also allowed the evaluation of the local influence on the overall measured noise brought by other noise sources. This was made possible by comparing the differences between the U-SPB-derived model for road traffic noise with the L_den_ and L_night_ measured values. The model derived from U-SPB parameters for each stretch was applied to Equation (1), taking into account measured traffic flows. The comparison is reported in the present chapter for four stretches among those laid in the project (note that ante-operam values for stretches 2 and 4 were unavailable, so their contribution was eliminated from the present work). Table 11 and Table 12 show that the model was able to sufficiently predict the measured level with small differences both ante- and post-operam. In terms of noise mitigation action, its efficiency was also demonstrated since almost all the noise was due to the traffic on the main road, i.e., the one where the noise mitigation was implemented. At night, some differences were seen for stretches 3 and 6, and they might be explained by the presence of a T intersection and a parking area, respectively.

## 6. Discussion and Conclusions

Evaluating the efficacy of low-noise surfaces has started to be a highly demanded activity since the publication of the European minimum environmental criteria. As a consequence, the measurement procedure involved deserves the highest degree of confidence and should be applicable in all contexts. Among the different methods, the Statistical Pass-By (SPB-ISO) method evaluates road traffic noise by means of microphone and speed acquisitions at the roadside in real traffic flow conditions. The original measurement setup does not allow its application in urban areas, which is where most inhabitants live and, thus, where the noise mitigation would have a larger effect.

The present paper reported a method for on-field acquisitions, named the Urban Pass-By (U-SPB) method, which adapts the SPB-ISO to urban context. The event detection and their extraction are performed in lab, as well as the consequent analysis. The evolution the method represented allows the performance of measurements of vehicle pass-bys without the presence of operators, with a consequent saving of man hours. The method also uses the good achievements of previous attempts to adapt the method, such as:

- The use of higher acoustic sensor position and the use of an SEL indicator instead of L_Amax_, as suggested in the HARMONOISE/IMAGINE projects;
- The use of statistical data binning before fit, as in the LEOPOLDO project.

The testing, validation and application of U-SPB were based on multiple measurement campaigns performed for different sites and road conditions. The results of the procedure were in good agreement with the standard ones, with the additional advantage of needing a reduced time to produce them. In fact, as an unattended methodology, U-SPB can use data acquired even during the night and then reduce the overall time required for monitoring. Furthermore, the availability of night noise and traffic data allows the estimation of long-term L_den_ from long-term traffic counts or statistics, without the need for long noise measurement campaigns, and the evaluation of the presence of other sources influencing local disturbance. Such an estimation is more than a noise model as it is calibrated on site. 

It must be pointed out that the procedure can be used as before/after evaluation of mitigation measurement, allowing the comparison of levels measured in the same place in different traffic conditions. However, attention should be paid to possible factors influencing results, such as long queues, which might cause category mismatching, and temperature [25] and, more generally, seasons. Indeed, the predominance of specific vehicle categories such as mopeds can influence results when looking only at SPBI.

U-SPB can also be used to estimate the contribution of different sources to the overall measured sound level in cases where more than one road source is present. For this eventuality, traffic flows for each of the investigated roads are required. The same approach can be extended to the traffic composition evaluation, leading U-SPB to also be suitable for pointing out whether the noisiness is mainly due to light or heavy traffic or no traffic at all. In this regard, the method results are a valid support in driving the noise mitigation evaluation. Further applications of the method on different road types will allow the establishment of a sound methodology for better event detection and feature extraction; in particular, a possible improvement would be to use more precise methods such as the ones proposed in [24] to distinguish between vehicle categories and to find a specific minimum separation time interval for road type. 

Finally, while extending the evaluation of the efficiency of low-noise laying in an urban context, U-SPB combines the possibility of estimating long-term noise levels and traffic information for other environmental noise purposes, such as noise mapping and action planning, proving to be a useful tool in environmental noise management.

Further developments of the method could also test the ability to tune conventional prediction models to the local fleet based on U-SPB measurements. Application can be crucial in specific contexts where the fleet is different from the one assumed in the conventional model, i.e., a predominance of old vehicles or of electric vehicles. 

## Figures and Tables

**Table 1:** Summary of SPB method adaptations mentioned in the paper and their characteristics.

	SPB-ISO	SPB-HI	SPB-L	U-SPB
**Reference**	ISO/DIS 11819-1 [10]	HARMONOISE/IMAGINE [35,36]	LEOPOLDO [31,32]	NEREIDE [29]
**Parameter**	L_Amax_	SEL	SEL	SEL
**Microphone heights**	1 mic at 1.2 m height	2 mics at 1.2 m and 3 m height	2 mics at 1.2 m and 3 m height	1 mic at 4 m height
**Measurement distance from road lane**	7.5 m	7.5 m	7.5 m	3–15 m
**Measurement conditions**	Attended	Attended	Attended	Unattended
**Events triggering**	On site	On site	On site	In lab by use of counter traffic
**Fitting** **technique**	Linear fit	Linear fit	Binning linear fit	Binning linear fit

**Table 2:** Summary of measurement campaigns.

Step	Code	Site	Methods	Measurement Conditions	Results
Testing	T0	SR 439 Capannori (LU)	U-SPB	Extra urban road, low-noise surface, data previously acquired	Feasibility confirmed
Testing	T1	SP 224 Marina di Pisa (PI)	U-SPB, SPB-L	Urban road, worked surface, data acquired at roadside day and night, with 6 h controlled situations	Calculated parameter chosen and comparison test performed and passed
Validation	V	Viale Togliatti Sesto Fiorentino (FI)	U-SPB, SPB-L	Urban road, low-noise surface, full measurement protocol applied	Validation completed
Application	A	SR 439 Massarosa (LU)	U-SPB	Extra urban road, ante-/post-operam measurements	Surface evaluated

**Table 3:** Coefficients of the fit of data measured in T0. *a* and *b* units are dB(A).

	Near Lane			Far Lane		
Metric	a±da	b±db	$\chi^{2}$	a±da	b±db	$\chi^{2}$
SEL	69.5 ± 0.4	19.2 ± 4.5	0.42	68.6 ± 0.4	18.0 ± 5.6	0.10
L_Aeq_	62.8 ± 0.4	25.8 ± 4.6	0.69	60.5 ± 0.4	25.2 ± 5.7	0.23
L_Amax_	66.8 ± 0.4	25.2 ± 4.5	0.78	64.7 ± 0.4	26.4 ± 5.7	0.42

**Table 4:** Results with the morning common sample acquired in T1 for light vehicles. *a* and *b* units are dB(A).

Lane	Coefficient	Monitoring Station	Mic 1.2 m	Mic 3 m
**SEL**				
near	a±da	75.1 ± 0.6	72.6 ± 0.6	75.4 ± 0.6
	b±db	22.8 ± 4.5	22.3 ± 4.5	21.6 ± 4.5
far	a±da	76.3 ± 0.5	76.4 ± 0.6	76.4 ± 0.6
	b±db	18.9 ± 5.4	20.5 ± 5.4	20.2 ± 5.4
**L_Aeq_**				
near	a±da	68.7 ± 0.6	67.2 ± 0.6	69.6 ± 0.3
	b±db	31.3 ± 4.5	30.1 ± 4.5	29.6 ± 1.2
far	a±da	69.2 ± 0.5	71.3 ± 0.5	70.7 ± 0.6
	b±db	26.5 ± 5.4	28.6 ± 5.5	28.7 ± 5.5
**L_Amax_**				
near	a±da	72.7 ± 0.6	70.9 ± 0.6	73.1 ± 0.6
	b±db	30.4 ± 4.5	29.3 ± 4.9	28.6 ± 4.9
far	a±da	73.3 ± 0.5	75.6 ± 0.5	75.3 ± 0.6
	b±db	26.3 ± 5.3	29.1 ± 5.4	26.1 ± 5.5

**Table 5:** SEL fits comparison for night U-SPB and SPB in T1. *a* and *b* units are dB(A).

Category	Lane	Parameter	U-SPB	SPB-L	
			Monitoring Station	Mic. 1.2 m	Mic. 3 m
Light	near	a±da	75.1 ± 0.5	72.6 ± 0.6	75.2 ± 0.6
		b±db	20.0 ± 4.4	22.3 ± 4.5	21.6 ± 4.5
		$\chi^{2}$	0.100	0.228	0.082
	far	a±da	75.7 ± 0.6	76.4 ± 0.6	76.1 ± 0.6
		b±db	23.8 ± 4.5	20.5 ± 5.4	20.2 ± 5.4
		$\chi^{2}$	0.215	0.371	0.252
Two wheelers	near	a±da	76.6 ± 1.1	72.2 ± 0.8	73.9 ± 0.7
		b±db	20.2 ± 11.0	15.2 ± 5.4	15.7 ± 5.4
		$\chi^{2}$	94	75	74
	far	a±da	76.4 ± 0.7	74.3 ± 1.0	73.1 ± 1.0
		b±db	20.4 ± 5.7	30.1 ± 12	30.9 ± 12
		$\chi^{2}$	26	61	61
Heavy	near	a±da	78.2 ± 1.0	74.4 ± 0.5	76.9 ± 0.4
		b±db	22.4 ± 8.4	46.5 ± 8.4	44.2 ± 6.3
		$\chi^{2}$	107	17	10
	far	a±da	80.8 ± 0.7	78.4 ± 1.2	78.2 ± 1.3
		b±db	17.0 ± 3.0	34.5 ± 16	32.1 ± 17
		$\chi^{2}$	44	52	55

**Table 6:** Number of analyzed pass-bys in T1 (night dataset and SPB-L).

Category	Lane	No. of Events SPB-L	No. of Events U-SPB
Light	near	114	129
	far	102	98
Two wheelers	near	13	15
	far	11	12
Heavy	near	10	15
	far	11	20

**Table 7:** Number of analyzed pass-bys in V.

Category	Lane	No. of Events SPB-L	No. of Events U-SPB
Light	near	65	120
	far	35	114
Two wheelers	near	13	31
	far	9	34
Heavy	near	8 two axes + 3 multiple axes	12 two axes + 10 multiple axes
	far	8 two axes	13 two axes + 12 multiple axes

**Table 8:** SEL fit comparison for night U-SPB and SPB-L in V. *a* and *b* units are dB(A).

Category	Lane	Parameter	Monitoring Station	Mic 1.2 m
Light	near	a±da	69.3 ± 0.5	69.5 ± 0.6
		b±db	17.7 ± 4.0	14.7 ± 9.3
		$\chi^{2}$	1.2	0.5
	far	a±da	68.5 ± 0.4	69.0 ± 0.8
		b±db	17.3 ± 4.6	14.5 ± 10.8
		$\chi^{2}$	3.6	0.2
Two wheelers	near	a±da	73.9 ± 1.2	73.4 ± 0.6
		b±db	8.7 ± 10.9	9.3 ± 4.6
		$\chi^{2}$	0.2	40
	far	a±da	74.5 ± 0.8	73.5 ± 0.9
		b±db	21.7 ± 7.4	15.7 ± 9.5
		$\chi^{2}$	2.4	44
Heavy	near	a±da	72.0 ± 0.9	74.3 ± 1.5
		b±db	11.5 ± 10.3	9.3 ± 4.6
		$\chi^{2}$	108	183
	far	a±da	70.6 ± 1.0	73.5 ± 0.9
		b±db	15.8 ± 9.4	15.7 ± 9.5
		$\chi^{2}$	113	39

**Table 9:** SPBI comparison for U-SPB and SPB standard in V. Values in dB(A).

Lane	SPBI (U-SPB)	SPBI (SPB-L)
near	71.1 ± 3.5	70.6 ± 12.5
far	70.4 ± 7.6	69.7± 5.4

**Table 10:** Noise levels according to measurements and pass-by model.

Noise Metric	L_meas_ (dB(A))	L_model_ (dB(A))	L_meas_ − L_model_ (dB(A))
L_D_	62.0	61.8	0.2
L_E_	59.1	58.3	0.8
L_N_	54.0	52.4	1.6
L_DEN_	63.0	62.1	0.9

**Table 11:** L_den_ values ante- and post-operam in Massarosa. Levels in dB(A).

	Ante-Operam			Post-Operam		
Stretch	L_meas_	L_model_	L_meas_ − L_model_	L_meas_	L_model_	L_meas_ − L_model_
1	67.8	66.5	1.3	64.8	64.5	0.3
3	70.5	69.1	0.6	65.5	63.6	1.9
5	72.7	72.4	0.3	69.4	68.5	0.9
6	71.8	71.0	0.8	67.6	67.2	0.4

**Table 12:** L_n_ values ante- and post-operam in Massarosa. Levels in dB(A).

	Ante-Operam			Post-Operam		
Stretch	L_meas_	L_model_	L_meas_ − L_model_	L_meas_	L_model_	L_meas_ − L_model_
1	59.4	58.1	1.3	56.7	56.7	0.0
3	62.6	61.2	1.4	57.4	55.8	1.6
5	64.8	64.1	0.7	61.1	60.1	1.0
6	63.8	62.4	1.4	59.7	59.1	0.6
